# Cognitive and behavioral associated changes in manifest Huntington disease: A retrospective cross‐sectional study

**DOI:** 10.1002/brb3.2151

**Published:** 2021-06-10

**Authors:** Simone Migliore, Giulia D’Aurizio, Sabrina Maffi, Consuelo Ceccarelli, Giovanni Ristori, Silvia Romano, Anna Castaldo, Caterina Mariotti, Giuseppe Curcio, Ferdinando Squitieri

**Affiliations:** ^1^ Huntington and Rare Diseases Unit Fondazione IRCCS Casa Sollievo della Sofferenza Hospital San Giovanni Rotondo Italy; ^2^ Department of Biotechnological and Applied Clinical Sciences University of L'Aquila L'Aquila Italy; ^3^ Italian League for Research on Huntington and Related Diseases (LIRH) Foundation Rome Italy; ^4^ Department of Neuroscience, Mental Health and Sensory Organs Faculty of Medicine and Psychology Centre for Experimental Neurological Therapies S. Andrea Hospital Sapienza University Rome Italy; ^5^ Department of Medical Genetics and Neurogenetics Fondazione IRCCS Istituto Neurologico Carlo Besta Milan Italy

**Keywords:** behavioral symptoms, cognitive deficit, dysexecutive syndrome, HD progression, Huntington disease

## Abstract

**Introduction:**

Behavioral and cognitive changes can be observed across all Huntington disease (HD) stages. Our multicenter and retrospective study investigated the association between cognitive and behavioral scale scores in manifest HD, at three different yearly timepoints.

**Methods:**

We analyzed cognitive and behavioral domains by the Unified Huntington's Disease Rating Scale (UHDRS) and by the Problem Behaviors Assessment Short Form (PBA‐s), at three different yearly times of life (t0 or baseline, t1 after one year, t2 after two years), in 97 patients with manifest HD (mean age 48.62 ± 13.1), from three ENROLL‐HD Centers. In order to test the disease progression, we also examined patients’ motor and functional changes by the UHDRS, overtime.

**Results:**

The severity of apathy and of perseveration/obsession was associated with the severity of the cognitive decline (*p* < .0001), regardless of the yearly timepoint. The score of irritability significantly and positively correlated with perseveration errors in the verbal fluency test at t0 (*r* = .34; *p* = .001), while the psychosis significantly and negatively correlated with the information processing speed at t0 (*r* = −.21; *p* = .038) and significantly and positively correlated with perseveration errors in the verbal fluency test at t1 (*r* = .35; *p* < .0001).

The disease progression was confirmed by the significant worsening of the UHDRS‐Total Motor Score (TMS) and of the UHDRS‐Total Functional Capacity (TFC) scale score after two‐year follow‐up (*p* < .0001).

**Conclusion:**

Although the progression of abnormal behavioral manifestations cannot be predicted in HD, the severity of apathy and perseveration/obsessions are significantly associated with the severity of the cognitive function impairment, thus contributing, together, to the disease development and to patients’ loss of independence, in addition to the neurological manifestations. This cognitive‐behavior pattern determines a common underlying deficit depending on a dysexecutive syndrome.

## Graphical abstract

Severity of apathy and perseveration/obsessions are significantly associated with the severity of the executive function impairment. This association may represent an additional key factor contributing to the severity of Huntington disease course. This cognitive‐behavior pattern determines a common underlying deficit attributable to a dysexecutive syndrome.

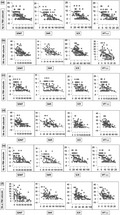

## 1 INTRODUCTION

Huntington disease (HD) is a rare, genetic, severely progressive, neurodegenerative illness manifesting through a combination of behavioral and neurological symptoms, that can start at any age. The cause is an expanded CAG repeat mutation which is detectable by a genetic test, before the first symptoms appear. The age of onset depends on the presence of neurological manifestations, that is, clinical motor and/or cognitive signs and/or behavioral symptoms that have an impact on life (Reilmann et al., 2014). The first symptoms usually manifest in adulthood, even though a pediatric (Fusilli et al., 2018) or a late onset are also possible (Reilmann et al., 2014). The progressive neurological manifestations are characterized by a movement disorder (e.g., choreic and dystonic movements, poor balance and coordination, Parkinsonism) and by a cognitive decline (e.g., executive dysfunction and memory failures), associated with behavioral abnormalities (e.g., depression, suicidal ideation, irritability, obsessions and perseveration, psychosis). All together, these progressive clinical manifestations lead to high disability, cachexia, dementia, and death (Reilmann et al., 2014).

Even though all the first motor, behavioral, and cognitive onset symptoms are extremely subtle and may mislead the correct clinical diagnosis (Pascu et al., 2015), motor symptoms tend to be more visible in the prodromal HD phase (i.e., the life time which is close to motor onset symptoms) and may help tracking the disease progression (Marder et al., 2000). Behavioral changes are instead nonspecific because overlap to a number of different behavioral conditions and vary largely and unpredictably across all HD stages (Eddy et al., 2016). In addition, even though cognition shows abnormalities since many years before the movement disorder becomes visible (Tabrizi et al., 2013), only the early cognitive impairment is measurable with internationally validated scales in late premanifest and early manifest HD (Stout et al., 2011).

The early impairment of cognition in HD includes abnormalities in the emotional recognition (Stout et al., 2011), in the theory of mind (Eddy & Rickards, 2015), and in the executive functions (e.g., working memory, task switching, inhibitory control, interference resolution, fluency, planning, and problem‐solving abilities) (Migliore et al., 2019; You et al., 2014). Besides the cognitive decline, the behavioral symptoms are also documented and may include personality changes due to the inability to control the mood (Eddy et al., 2016). The mood alteration includes anxiety, depression and suicidality, irritability bursts and aggressiveness, obsessions, and perseveration. More rarely, the psychosis with delusions and hallucinations may contribute to HD psychopathology. The symptom apathy also represents a common behavioral disturbance and is considered among the few behavioral changes worsening as the disease progresses (Tabrizi et al., 2013). Previous studies highlighted a relationship between behavioral abnormalities and other clinical variables, both in manifest and in premanifest HD stages. For example, some studies (Baudic et al., 2006; Thompson et al., 2002) showed that apathy was directly related to the cognitive and motor score worsening in manifest HD. Other studies (Duff et al., 2010; Smith et al., 2012) confirmed this correlation also since prodromal HD, thus disclosing a possible relationship between some behavioral domains (i.e., apathy, depression, disinhibition) and the frontal cognitive skills.

The cognitive and behavioral abnormalities are likely related to each other as a biological consequence of gangliathalamocortical circuit dysfunction and may be reduced by silencing the mutated gene in animal models (Yamamoto et al., 2000). The cognitive abnormalities, such as deficit in social cognition (i.e., emotion recognition and theory of mind), and abnormal behaviors, such as incongruity, apathy, irritability, inflexibility, and intransigence in social situations, appear across all HD stages (Bora et al., 2016), before the occurrence of dementia.

However, even though the progressive decline of cognition and the unpredictable appearance of behavioral symptoms severely impair patients’ independence and represent the greatest stressor for HD families, especially for the caregivers (Duff et al., 2007), there is no fully documented relationship between behavioral and cognitive abnormalities and their change over time. As a consequence, reliable quantification of measures combining both these patterns of symptoms is still missing.

By our multicenter and retrospective study, we aimed to primarily explore a potential relationship between cognitive alterations and behavioral symptoms, that were measured in manifest HD. Compared to all the previous studies, our analysis included the correlation between the several behavioral and cognitive scores, which we measured, overtime, at three different yearly timepoints during the patients’ life (t0 or baseline, t1 after one year, t2 after two years). In addition, to document the neurological progression of HD overtime and across the yearly timepoints, we also analyzed the worsening of motor impairment together with the loss of independence, as a secondary end point.

## 2 METHODS

### 2.1 Patients’ population

Starting from a sample of 177 patients recruited in three Enroll‐HD Centres in Italy, for example, LIRH Foundation (Rome), IRCCS Istituto Neurologico Carlo Besta (Milan); Sant’Andrea University Hospital (Rome)—we selected 97 patients based on the following inclusion criteria: (1) neurological age at onset after age 20; (2) Unified Huntington's Disease Rating Scale (UHDRS)—Total Motor Score (TMS) >10 and Diagnostic Confidence Level (DCL) of 4 at baseline; (3) UHDRS‐Total Function Capacity (TFC) ≥ 3 at baseline; and (4) no history of neurological conditions, other than HD and of substance dependence or of developmental disorder affecting cognition, at the baseline assessment. All patients were assuming small doses of neuroleptics (olanzapine 2.5–10 mg daily, aripiprazole 2.5–10 mg daily, quietiapine 25–50 mg daily, pimozide 2–4 mg daily, haloperidole 0.5–1 mg daily) and benzodiazepines (lorazepam, bromazepam, alprazolam 0.5–2 mg daily). HD was genetically confirmed in all cases (all with expansion ≥40 CAG repeats).

### 2.2 Measures

All patients were assessed by the UHDRS (Unified Huntington's Disease Rating Scale, 1996) and by PBA‐s (Callaghan et al., 2015) at baseline (time 0; t0) and at 1‐year (time 1; t1) and 2‐year (time 2; t2) follow‐up, by health professionals with expertise in HD, in accordance with ENROLL‐HD guidelines (Landwehrmeyer et al., 2016) and after signed informed consent.

### 2.3 UHDRS: motor, cognitive, and functional assay

UHDRS included motor, cognitive, and functional domains (e.g., TMS or TFC, Independence Scale or IS, and Functional Assessment or FA). The TMS is a standardized and validated assay consisting of 31 items rating the motor impairment from 0 to 4 with a score of 0 indicating no abnormalities and 4 indicating the most severe impairment (Unified Huntington's Disease Rating Scale, 1996). The TFC is a standardized and validated assay of overall function in HD. The TFC rates individuals’ function on the following domains: occupation, handling finances, domestic chores, and activities of daily living and ranges from 13 (normal function) to 0 (complete loss of function) (Unified Huntington's Disease Rating Scale, 1996). The cognitive evaluation includes (1) Categorical Verbal Fluency Test (VFT) in which participants have to produce as many words as possible from a semantic category (usually animals) in a given time (60 s); (2) Stroop Color Reading Test (SCR) in which participant is requested to name as many color as possible in 45 s of randomly presented tokens (three colors—blue, red, green) and Stroop Word Reading Test (SWR) where the participants read as many words as possible in 45 s of randomly presented tokens (three color words—blue, red, green) printed in black ink; and (3) The Symbol Digit Modality Test (SDMT), in the written response format, requires the participants to use a coded key to match nine numerical digits with nine symbols in 90 s. Participants were given 10 practice items before starting the test. In all cases, lower are the scores, more pronounced is the cognitive decline.

### 2.4 Problem Behavioral Assessment‐short form (PBA‐s): behavioral assay

The PBA‐s test rates the behavioral changes by an 11‐item semistructured interview specifically designed to cover a wide range of behavioral symptoms related to HD (Kingma et al., 2008). This scale is usually administered in the presence of the caregiver/companion and rates the severity (0: absent, 1: questionable, 2: mild, 3: moderate, and 4: severe) and the frequency (0: never/ almost never, 1: seldom, 2: sometimes, 3: frequently, and 4: daily/almost daily for most or all the day) of scores of depressed mood, suicidal ideation and anxiety (Depression subscore: Dep‐sc), irritability and aggressive behavior (Irritability subscore: Ir‐sc), apathy and disoriented behavior (Apathy subscore: Ap‐sc), perseverative thinking and obsessive–compulsive behaviors (Perseverative/Obsessive subscore: Db‐sc), paranoid thinking and hallucinations (Psychosis subscore: Pt‐sc), in accordance with ENROLL‐HD guidelines. The PBA score ranges between 0 and 176 with the higher scores associated with the most severe behavioral abnormalities.

### 2.5 Ethical approval

This study conforms with World Medical Association Declaration of Helsinki and was approved by the Institutional Review Board of the coordinator site (LIRH Foundation, prot. number 102/14, approved in date 28/05/2014); as a consequence, all participants signed an informed consent.

### 2.6 Statistical analysis

In order to assess differences in both the motor and functional scores, as well as in the PBA‐s and cognitive performances at the three timepoints, a different one‐factor repeated‐measure analysis of variance (ANOVA) was performed for TMS, TFC, PBA subscales (Dep‐sc; Ir‐sc Ap‐sc; Db‐sc; Pt‐sc), cognitive test (VFT, correct response and perseveration; SCR, correct responses; SWR, correct responses; SDMT, correct responses) with Time (t0, t1, and t2 timepoint interval) as within factor. Alpha level was fixed to ⩽0.05, and in case of significant main effects, post hoc comparisons (Bonferroni's multiple comparisons) were carried out. To assess the relationships between PBA‐s subscale scores and cognitive functions an exploratory Pearson's correlation analysis was run using Dep‐sc, Ir‐sc, Ap‐sc, Db‐sc, Pt‐sc, VFT (correct response and perseveration), SCR (correct responses), SWR (correct responses), and SDMT (correct responses) as dependent variables. The correlation analysis was repeated for each of the three timepoints (t0, t1, and t2). Finally, we evaluated the potential correlation between the CAG mutation length and all cognitive and behavior variables. Alpha level was fixed to ⩽0.05. All statistical analyses were performed using IBM SPSS Statistics for Macintosh, version 25.0 (IBM Corp).

## 3 RESULTS

### 3.1 Patient characteristics

Our population of 97 patients included 39 females and 58 males, mean age of 48.62 ± 13.1 years (range 26–79 years), education level of 11.82 ± 4.27 years (range 5–18 years), mean age of neurological onset manifested at 45.18 ± 12.3 years (range 22–72 years), and mean expanded CAG repeat number of 44.32 ± 3.8 (range 40–57 CAG repeats).

### 3.2 Correlation between behavioral and cognitive scores

**FIGURE 1:**
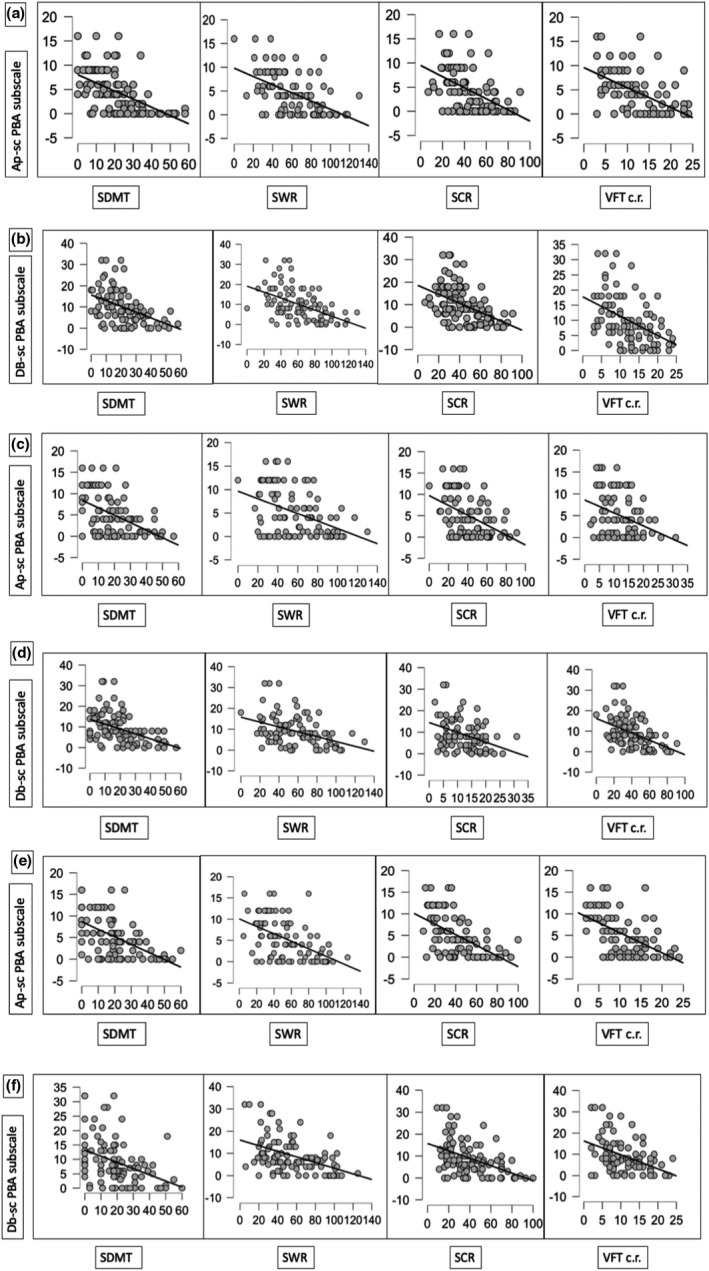
Main correlation Matrixs’ plots

Figure 1 shows the main significant correlation between all cognitive scores and apathy and between all cognitive scores and the perseveration/obsession domains. Table 1 shows the correlation between the cognitive and behavioral scores observed at each timepoint.

**TABLE 1:** Pearson's r (and related level of significance) between behavioral subscales and all cognitive domains at three different timepoints. Pearson's *r* (and related level of significance) between PBA‐subscore and SDMT, VTF c.r., VTF pe, SCR, SWR at (a) T0; (b) T1; (c) T2

	Dep		Ir		Pt		Ap		Db	
	*r*	*p*	*r*	*p*	*r*	*p*	*r*	*p*	*r*	*p*
(a)										
SDMT	−.14	.177	−.18	.053	−.18	.082	−.54	.**000**	−.46	.**000**
VTF c.r.	−.05	.595	−.09	.364	−.15	.152	−.55	.**000**	−.45	.**000**
VTF pe	.03	.766	.34	.**001**	.04	.684	.15	.152	.07	.485
SCR	−.04	.693	−.13	.194	−.18	.084	−.49	.**000**	−.46	.**000**
SWR	−.01	.337	−.17	.100	−.21	.**039**	−.54	.**000**	−.49	.**000**
(b)										
SDMT	−.18	.090	−.09	.407	−.19	.063	−.51	.**000**	−0.46	.**000**
VTF c.r.	−.14	.181	−.07	.460	−.11	.289	−.36	.**000**	−0.37	.**000**
VTF pe	−.04	.662	.04	.689	.35	.**000**	−.01	.98	0.04	.719
SCR	−.08	.417	−.08	.437	−.19	.067	−.45	.**000**	−0.47	.**000**
SWR	−.15	.135	−.05	.625	−.05	.638	−.43	.**000**	−0.43	.**000**
(c)										
SDMT	−.05	.593	−.11	.300	−.15	.139	−.55	.**000**	−0.46	.**000**
VTF c.r.	.00	.966	−.16	.125	−.19	.064	−.52	.**000**	−0.44	.**000**
VTF pe	−.08	.439	−.01	.916	.15	.150	.02	.843	−0.04	.705
SCR	−.04	.717	−.08	.461	−.21	.053	−.54	.**000**	−0.45	.**000**
SWR	−.11	.274	−.11	.280	−.16	.115	−.53	.**000**	−0.45	.**000**

Abbreviations: Ap, apathy PBA‐s subscale; Db, obsessions/perseverative thinking PBA‐s subscale; Dep, depressed mood PBA‐s subscale; Ir, irritability PBA‐s subscale; Pt, delusions/paranoid thinking PBA‐s subscale; SCR, Stroop Color Reading Test; SDMT, Symbol Digit Modality Test.; SWR, Stroop Word Reading Test; VTF c.r., Categorical Verbal Fluency Test. correct responses; VTF pe, Categorical Verbal Fluency Test. Perseverations.

Statistical significance is highlighted in bold.

At t0, the analysis showed a significant negative correlation (*p *< .0001) between Ap‐sc and SDMT (*r *= −.54), VFT correct responses (*r *= −.55), SWR (*r *= −.54), and SCR (*r *= −.5) (Figure 1, panel A and B, Table 1a). We also found a significant negative correlation (*p* < .0001) between Db‐sc and SDMT (*r *= −.46), VFT correct responses (*r *= −.45), SWR (*r *= −.5), and SCR (*r *= −.46). Ir‐sc score significantly and positively correlated with VFT perseveration errors (*r *= .34; *p* = .001). Pt‐sc significantly and negatively correlated with SWR (*r *= −.21; *p* = .038).

At t1, the analysis showed a significant negative correlation (*p* < .0001) between Ap‐sc with SDMT (*r *= −.51), VFT correct responses (*r *= −.36), SWR (*r *= −.43), and SCR (*r *= −.45). Db‐sc showed a significant negative correlation (*p* < .0001) with SDMT (*r *= −.46), VFT correct responses (*r *= −.37), SWR (*r *= −.43), and SCR (*r *= −.47), while Pt‐sc showed a significant positive correlation with VFT perseveration errors (*r *= .35; *p* < .0001) (Figure 1, panel C and D, Table 1b).

At the t2, the analysis showed a significant negative correlation (*p* < .0001) between Ap‐sc and SDMT (*r *= −.55), VFT correct responses (*r *= −.52), SWR (*r *= −.53) and SCR (*r *= −.54), as well as between Db‐sc and SDMT (*r *= −.46), VFT correct responses (*r *= −.44), SWR (*r *= −.45), and SCR (*r *= −.45; Figure 1, panel E and F, Table 1c).

The statistical significance was not influenced by the CAG mutation size.

### 3.3 Motor and functional assay

The TMS significantly changed in dependence of the time, after 2‐year follow‐up (*F*_2.188_ = 68.32; *p* < .0001; $\eta_{p}^{2}$ = 0.42), indicating a TMS at t2 (*M* = 43.67, *SD* = 22.8) higher than at t1 (*M* = 38.82, *SD* = 21.77) and then at t0 (*M* = 34.56, *SD* = 20.6). Bonferroni's multiple comparisons revealed significant differences among all timepoints (*p* < .0001). The TFC also significantly changed in dependence of the time after 2‐year follow‐up (*F*_2.192_ = 71.85; *p* < .0001; $\eta_{p}^{2}$ = 0.43), indicating a TFC at t2 (*M* = 7.72, *SD* = 3.8) lower than at t1 (*M* = 8.42, *SD* = 3.65) and than at t0 (*M* = 9.14, *SD* = 3.25). The post hoc tests revealed significant differences between timepoints (*p* < .0001) (Table 2).

**TABLE 2:** Mean and standard deviation of patients’ scores across the clinical variables, levels of significance one‐factor repeated‐measure ANOVA, and post hoc tests between behavioral subscales and all cognitive domains and clinical variables at three different timepoints

Clinical assay	Clinical variables	T0	T1	T2	*p*	*p* (T0 vs. T1)	*p* (T0 vs. T2)	*p* (T1 vs. T2)
PBA‐s Behavioral subscales	Dep	7.08 ± 5.64	6.72 ± 6.2	5.58 ± 4.7	.038	1	0.07	0.07
	Ir	3.73 ± 4	3.9 ± 4.2	4.7 ± 6.5	.15	1	0.34	0.52
	Ap	4.43 ± 4.3	4.96 ± 4.9	5.23 ± 4.7	.13	0.51	0.18	1
	Db	9.85 ± 8.04	8.9 ± 7.2	9.03 ± 7.82	.31	0.35	0.87	1
	Pt	0.64 ± 2.14	0.56 ± 1.72	0.66 ± 2.01	.86	1	1	1
UHDRS cognitive measures	VTF c.r.	12.43 ± 5.7	12.24 ± 5.93	11.03 ± 5.23	<.0001	1	0.001	0.001
	VTF pe.	0.6 ± 0.92	0.7 ± 1.02	0.47 ± 0.94	.17	1	0.8	0.2
	SCR	43.94 ± 18.72	41.22 ± 19.1	39.7 ± 20.7	<.0001	0.006	0.002	0.38
	SWR	62.04 ± 26.7	58.94 ± 26.77	55 ± 28	<.0001	0.03	<0.0001	0.02
	SDMT	21.7 ± 13.86	20.32 ± 14.21	19.85 ± 14.66	.04	0.017	0.11	1
UHDRS motor and functional measures	TMS	34.56 ± 20.6	38.82 ± 21.77	43.67 ± 22.8	<.0001	<0.0001	<0.0001	<0.0001
	TFC	9.14 ± 3.25	8.42 ± 3.65	7.72 ± 3.8	<.0001	<0.0001	<0.0001	<0.0001

Abbreviations: Ap, apathy PBA‐s subscale; Db, obsessions/perseverative thinking PBA‐s subscale; Dep, depressed mood PBA‐s subscale; Ir, irritability PBA‐s subscale; NS, Not Significant.; Pt, delusions/paranoid thinking PBA‐s subscale; SCR, Stroop Color Reading Test; SDMT, Symbol Digit Modality Test; SWR, Stroop Word Reading Test; TFC, Total Function Capacity; TMS, Total Motor Score; VTF c.r., Categorical Verbal Fluency Test. correct responses; VTF pe., Categorical Verbal Fluency Test. Perseverations.

### 3.4 Behavioral assay

The PBA‐s scores showed a statistically significant main effect of Time only for Dep‐sc (*F*_2,192_ = 3.34; *p* = .038; $\eta_{p}^{2}$ = 0.034), revealing a score at t0 (*M* = 7.08, *SD* = 5.64) higher than at t1 (*M* = 6.72, *SD* = 6.2) and than at t2 (*M* = 5.58, *SD* = 4.7). The post hoc tests did not reveal a significant difference between timepoints (Table 2).

### 3.5 Cognitive assay

Regarding the cognitive performance, repeated‐measure ANOVA showed a statistically significant main effect for Time for VTF correct responses (*F*_2,190_ = 9.8; *p* < .0001; $\eta_{p}^{2}$ = 0.093), with scores at t0 (*M* = 12.43, *SD* = 5.7) higher than at t1(*M* = 12.24, *SD* = 5.93) and than at t2 (*M* = 11.03, *SD* = 5.23). With respect to SCR, a significant main effect was found for Time (*F*_2,192_ = 8.72; *p* < .0001; $\eta_{p}^{2}$ = 0.083), indicating a better performance at t0 (*M* = 43.94; *SD* = 18.72) than at t1 (*M* = 41.22; *SD* = 19.1) and than at t2 (*M* = 39.7, *SD* = 20.7). Regarding to SWR, a significant main effect was found for Time (*F*_2,192_ = 11.9; *p* < .0001; $\eta_{p}^{2}$ = 0.11), revealing a better performance at t0 (*M* = 62.04, *SD* = 26.7) than at t1(*M* = 58.94, *SD* = 26.77) and than at t2 (*M* = 55, *SD* = 28). Finally, with respect to SDMT a significant main effect was found for Time (*F*_2,186_ = 3.31; *p* = .04; $\eta_{p}^{2}$ = 0.034), showing a higher accuracy at t0 (*M* = 21.7, *SD* = 13.86) than at t1 (*M* = 20.32, *SD* = 14.2) and than at t2 (*M* = 19.85, *SD* = 14.66). The post hoc analyses were assessed for all cognitive scores; details are reported in Table 2.

## 4 DISCUSSION

Our cross‐sectional, retrospective, and multicenter study describes a significant association between UHDRS cognitive scores and the score of some specific behavioral changes measured by PBA‐s. In spite of a significant, time‐dependent, worsening of motor and functional progression during the 2‐year follow‐up, the behavioral changes remained unpredictable, thus confirming previously reported findings (Eddy et al., 2016). However, one original finding we describe here is the association between the severity of some behavioral changes—that is, the items concerning the apathy and the perseveration/obsession symptom scores—and the score of cognitive functions worsening. Such correlation was time independent, thus suggesting a statistical association between the cognitive decline and the score of specific behavioral modifications at each timepoint. Some other behavioral changes, such as the irritability or features of psychosis (i.e., the persecutory behavior), showed a transitory correlation only at t0 (irritability and psychosis) and later in the course of HD, at t1 (i.e., the psychosis), thus highlighting an inconstant association between given behavioral and cognitive items. However, considering the potential effects of neuroleptics on HD progression (Tedroff et al., 2015), whether the effect of pharmacotherapy might have played a role by acting against some pattern of cognitive and behavioral symptoms, remains to be elucidated.

Even though the etiology of behavioral changes still requires additional studies and further confirmation and thus remains unclear, the involvement of brain cortical–striatal circuits may potentially explain, at least in part, the high prevalence of behavioral manifestations affecting HD patients (van Duijn et al., 2007). The same cerebral pathways are also implicated in determining different cognitive domains, especially the high‐level functions, such as the executive skills, that are specific abilities needed for complex, goal‐directed, behaviors and for adaptation to environmental changes and to the information processing speed (Lezak, 2004). In particular, the prefrontal cortex, that has reciprocal connections with subcortical brain areas, also represents a multimodal association brain cortical region with the task of information processing from various sensory modalities and integrates the different cognitive processes (Cummings, 1993). Our findings are therefore in line with the recent observations showing the clinical association of behavioral manifestations, such as the apathy and the irritability, with abnormal executive processes that start to decline since the prodromic HD stage of life (Martinez‐Horta et al., 2016; Misiura et al., 2019), and with striatum abnormalities (Misiura et al., 2019) or with disrupted cortical–striatal white‐matter tracts (De Paepe et al., 2019). It is therefore possible that some worsen behavioral scores are related to the cognitive decline, especially for it concerns the measures rating the executive impairment. It is also possible that both behavioral and cognitive patterns mirror a common underlying deficit attributable to a dysexecutive syndrome as a consequence of the disrupted brain cortical–striatal pathway and connectivity.

Our data are also in line with findings from other neurodegenerative diseases where depression (Rapp et al., 2006), or depression associated with apathy and agitation (Apostolova & Cummings, 2008), were prodromic to the cognitive decline in onset Alzheimer diseases (AD), or where apathetic symptoms correlated with worsening of memory scores and of executive dysfunction in AD (Robert et al., 2006) and in Parkinson disease (PD) (Grossi et al., 2013). All together, these observations lend support to the evidence of potential clinical association between some behavioral and cognitive pattern of symptoms in neurodegenerative diseases, with obvious implications with the impairment of specific cerebral abnormal pathways and with social cognitive deficits in most of the patients (Bora et al., 2016).

However, our study has limitations. Firstly, we have a small country and language‐specific sample size and we would need prospective confirmation with larger and multilingual cohorts. Secondly, we cannot exclude an effect of drugs, that is, the neuroleptics, although limited by the low doses which were used in our patients. Finally, we did not test whether and how the neuropathology contributed to the patterns of abnormalities because it was not among the aims of our study. Moreover, it would be also interesting to stratify the cohort according to age at onset or mutation length to test whether juvenile‐onset patients may differ in some correlations.

Despite these limitations, our research represents one of the few studies stating that cognition and behavior may be directly related and may influence each other overtime thus contributing, together, to the severity of HD, including the neurological progression and the loss of independence. The awareness of a correlation between behavioral and cognitive changes has potential obvious implications with the patients’ quality of life and with clinical and therapeutic interventions, including caregivers’ psychoeducational‐based strategies. Interestingly, this cognitive‐behavioral pattern, likely attributable to a dysexecutive syndrome, occurs also in other neurodegenerative diseases (i.e., AD and PD) and also contributes to the loss of independence in those pathologies (Godefroy et al., 2010).

Recently, composite measures including specific cognitive, motor, and functional items have been proposed to track premanifest (Long et al., 2017) and manifest HD (Schobel et al., 2017). Such composite measures are currently included as major endpoints in some phase‐3 clinical trials, such as the Roche‐Genentech Generation‐HD1 with *tominersen*, an experimental antisense‐drug therapy (Tabrizi et al., 2019). It would be worth taking into consideration some behavioral items, in addition to cognitive, motor, and functional scores, in the attempt to propose further composite measures.

## 5 CONCLUSION

Our findings highlighted a relationship between some worsen behavioral scores (i.e., apathy, obsessive/perseverative symptoms) and abnormalities in cognitive functions overtime, suggesting a probably common underlying deficit attributable to a dysexecutive syndrome, as a consequence of the frontal–subcortical cerebral pathway disruption. Moreover, our findings could be useful also to further explore cognitive and behavioral domains to test whether their abnormalities may offer new composite measures to improve symptoms prediction, to track HD progression, and to highlight potential correlation with neuropathological/imaging brain changes during time. Large observational studies, such as the multicenter Enroll‐HD platform (Landwehrmeyer et al., 2016), together with the analysis of new cognitive assessment tools imported by the study of other neurodegenerative diseases (Martinez‐Horta et al., 2020), may represent an opportunity to gain further insight into this devastating disease where multidisciplinary care is strongly requested.

## CONFLICT OF INTEREST

All authors declare no conflict of interest. All authors confirm agreement with the final version of the manuscript.

## AUTHORS CONTRIBUTION

SM and FS developed the study concept, contributed to the study design, and provided data interpretation. SM, SaM, CC, SR, GR, AC, and CM performed testing and data collection. GC and GD performed the data analysis and data interpretation. FS drafted the original draft. All authors provided revisions and approved the final version of the paper for submission.

### PEER REVIEW

The peer review history for this article is available at https://publons.com/publon/10.1002/brb3.2151.

## Data Availability

The data that support the findings of this study are available from the corresponding author upon reasonable request.
